# A non-optically active lake salinity dataset by satellite remote sensing

**DOI:** 10.1038/s41597-025-05686-2

**Published:** 2025-07-30

**Authors:** Mingming Deng, Ronghua Ma, Lixin Wang, Minqi Hu, Kun Xue, Zhigang Cao, Junfeng Xiong, Zhengyang Yu

**Affiliations:** 1 Key Laboratory of Lake and Watershed Science for Water Security, Nanjing Institute of Geography and Limnology, Chinese Academy of Sciences, Nanjing, 210008 China; 2 University of Chinese Academy of Sciences, Beijing, 100049 China; 3 University of Chinese Academy of Sciences, Nanjing, 211135 China; 4 School of Ecology and Environment, Inner Mongolia University, Hohhot, 010021 China

**Keywords:** Hydrology, Limnology

## Abstract

Water salinity characterizes the physicochemical properties of natural water, serving as an essential parameter for assessing lake water quality. However, the efficiency of remote sensing inversion of water salinity is limited as salinity is a non-optically active parameter, leading to the lack of a pixel-scale lake salinity dataset. Conventional function models based on salinity tracers or single lakes have low regional applicability, while machine learning algorithms can effectively capture the nonlinear relationship between radiance and salinity, providing large-scale inversion opportunities. Our study constructed an extreme gradient boosting (XGB) salinity model, which was used to generate the Inner Mongolia lake salinity (IMSAL) dataset with Sentinel-2 remote sensing reflectance. The IMSAL dataset contains 928 raster scenes with 10-meter spatial resolution for eight lakes from 2016 to 2024. Cross-validation and independent validation with measured and published literature-recorded salinities confirmed the good consistency and reliability. This dataset provides invaluable information on spatial patterns and long-term variations in lake salinity useful to prevent lake salinization and facilitate the lake management for sustainable ecosystem development.

## Background & Summary

Water salinity is crucial and maintains the stability of lake ecosystems as an essential parameter for evaluating water quality^1,2^, which affects the biological, physical, and chemical processes of lake ecosystems, including the survival and distribution of aquatic organisms, the utilization of water resources, and the carbon cycle of lakes^3–5^. Lake salinity exhibits a high spatiotemporal sensitivity under the impact of climate change and anthropogenic activity^6,7^, especially in arid and semi-arid regions^8,9^. However, field experiments do not provide adequate spatial detail, which makes it difficult to monitor salinity continuously.

Lake water quality information is frequently and extensively acquired by remote sensing in various bands of the electromagnetic spectrum, effectively compensating for the spatial dispersion and low-frequency problems of field surveys^10^. On board the Sentinel-2 satellite, the Multi-Spectral Instrument (MSI) sensor has high spatial resolution (10‒60 m), a high return frequency (5 days) with twin satellites, and considerable radiometric resolution (12 bit) with enhanced signal-to-noise ratios at 443 nm^11^. MSI images offer more detailed spatial characterization that is useful for water constituents monitored in small-scale inland lakes. It has been widely used to estimate the concentration of optically active constituents (OACs) and non-OACs, such as colored dissolved organic matter (CDOM), chlorophyll a (Chl*a*, μg/L), total nitrogen, and total phosphorus^12–14^. Lake salinity belongs to non-OACs and has no direct color signal and a complicated non-linear relationship with remote sensing reflectance *R*_*rs*_(λ) (units sr^−1^)^15^. The CDOM absorption coefficient (*a*_g_(λ), m^–1^) commonly served as a tracer of salinity in estuaries, while the correlation between *a*_g_(λ) and salinity cannot be significant in inland lakes^16,17^. In addition, previous studies have attempted to use Secchi Disk Depth (SDD, m) as an indirect parameter to retrieve lake salinity on the Tibetan Plateau^18^. However, this indirect inversion method relies on the accuracy of intermediate parameters that are not only regional-specific but also complicated in the retrieval process and inevitably produce errors. In order to minimize the error propagation, machine learning (ML) algorithms were used to process the nonlinear relationship between *R*_*rs*_(λ) and salinity by intricate structure and networks. ML is an innovative method to mine the information of non-OACs from satellite images and has been proved to be effective in inland waters by published studies, such as estimates of dissolved oxygen, particulate organic carbon, and dissolved organic carbon concentrations^19–21^. Apart from other non-OACs, regional-scale lake salinity estimates have always been challenged by diverse climatic and geographic conditions, as the complexity of lake hydrology, ion composition, and sources results in multiple magnitudes. Furthermore, the high diversity of Chl*a*, *a*_g_(λ), and suspended particulate matter (SPM, mg/L) causes optical complexity in inland water and has made the uniform salinity algorithm developed hard^17,22^. Therefore, the remote sensing of non-optically active salinity, which varies from place to place, needs to be effectively monitored, especially in arid and semi-arid areas where highly dynamic changes occur.

**Fig. 1:**
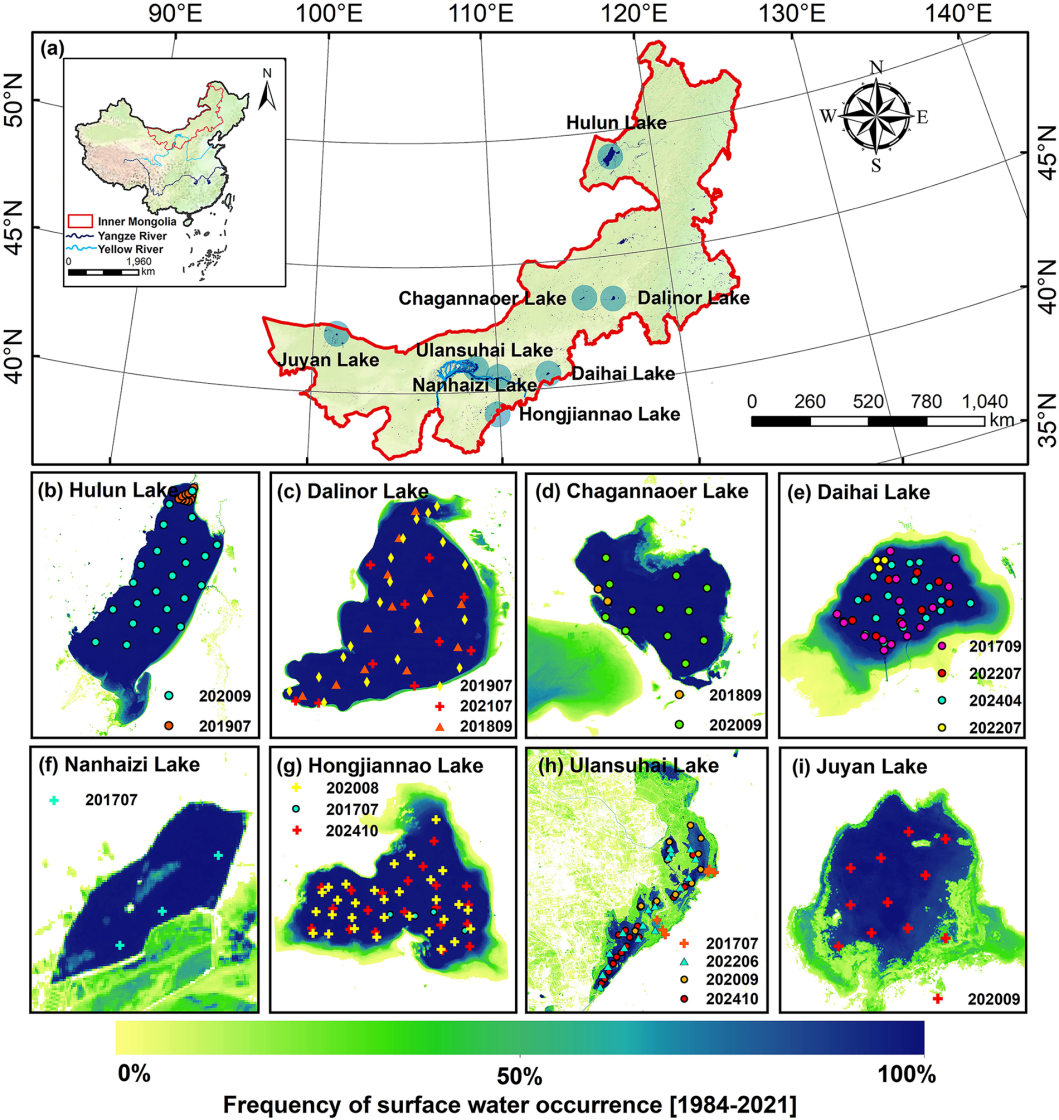
(**a**) Location of sampled lakes, (**b**–**i**) distribution of sampling points and frequency of water occurrence for eight lakes from east to west in Inner Mongolia. Surface water frequency data were from the Global Surface Water dataset (https://global-surface-water.appspot.com/)^59^.

Inner Mongolia is located in a typical arid and semi-arid transition zone, crossing most climatic regions from east to west in China (Fig. 1), with the development of various lake types, including freshwater (<1 g/L), brackish (1–3 g/L), and oligosaline (3–35 g/L) (Table 1)^23^. These lakes are critical shields for ecological security in northern China and provide valuable water resources to maintain ecosystem functions^24,25^. However, the long-term satellite monitoring of lake salinity is not present in the region and it is hard to reveal the spatiotemporal distribution. Consequently, the Inner Mongolia lake salinity (IMSAL) dataset for eight lakes over the past nine years was created based on Sentinel-2 MSI data and the XGB algorithm, which offers multi-scale (daily, annual, intra-annual season, and season) average records and pixel-scale spatial distributions of lake salinity. The purpose of this research is to (1) innovatively construct a 10 m spatial resolution salinity dataset using MSI images, (2) provide a dataset paradigm for other lakes to generate salinity datasets, and (3) share long-term salinity data to facilitate lake management and prevent lake salinization.

**Table 1:** Statistics of water quality parameters of sampled lakes.

Lake Name	Sample Points	Salinity (ppt)		SDD (m)	
		Mean ± S.D.	Range (Min-Max)	Mean ± S.D.	Range (Min-Max)
Hulun	35	0.78 ± 0.08	0.54–0.86	0.29 ± 0.02	0.26–0.33
Dalinor	42	6.42 ± 0.16	6.15–6.60	0.48 ± 0.06	0.36–0.54
Chagannaoer	15	0.86 ± 0.03	0.83–0.92	/	/
Daihai	56	13.56 ± 2.38	10.67–16.81	2.37 ± 1.10	0.63–4.80
Hongjiannao	54	6.34 ± 0.18	5.82–7.00	1.67 ± 0.29	0.70–2.20
Nanhaizi	3	1.39 ± 0.01	1.39–1.41	0.27 ± 0.02	0.24–0.29
Ulansuhai	53	1.72 ± 0.54	0.86–3.27	1.23 ± 0.32	0.24–2.20
Juyan	11	4.61 ± 0.11	4.53–4.93	/	/

## Methods

**Fig. 2:**
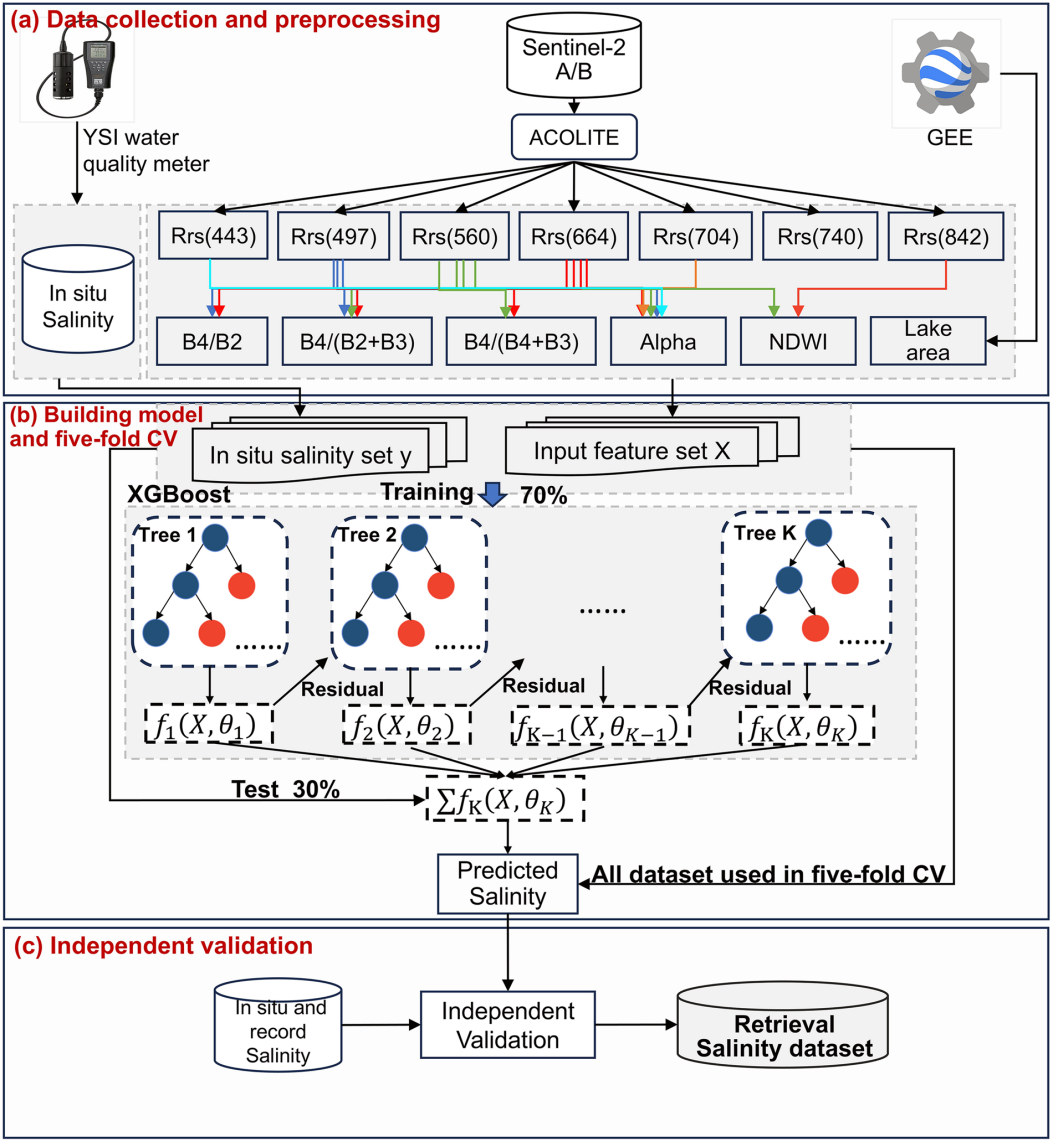
Overall working framework for producing salinity dataset from Sentinel-2 MSI images.

The IMSAL dataset contains salinity raster data from 2016 to 2024 for eight lakes in Inner Mongolia. Figure 2 overviews the workflow for salinity retrieval and dataset generation, which consists of three major modules. Module one is the data collection and preprocessing for *in situ* and MSI data, including field surveys, atmospheric correction, water extraction, and index construction. Module two is the construction of the XGB salinity retrieval model, testing, and five-fold cross-validation (CV). The final module is an independent validation by using the latest *in situ* and literature-recorded data.

### Field data collection

Field data were obtained through uniformly distributed *in situ* sampling and previous research in eight lakes from July 2017 to October 2024. All data were collected in spring (March to May), summer (June to August), and autumn (September to November), lacking winter data due to ice cover. A total of 318 data were collected: 229 from *in situ* measurements and 89 from published literature^26–37^. Usage details about literature record salinity are shown in Table 2. These data were divided into three datasets, *Dataset one* contains 229 data pairs for model training, testing, and five-fold CV, with a ±3 days matching interval between field data and MSI images. *Dataset two* consists of 69 latest field-measured and literature records salinity data, which were used for independent validation of the IMSAL. *Dataset three* comprises 62 *in situ* samples were collected concurrent with Sentinel-2 overpass (±6 hours) and measured parameters including spectrum, salinity (ppt), Chl*a*, *a*_g_(λ), SPM, and SDD, which were applied to assess *R*_*rs*_(λ). Salinity was measured at the lake surface (depth <0.5 m) by a calibrated YSI water quality meter (YSI, Inc.). Surface water samples collected were stored in a lightproof box and returned to the laboratory immediately to measure Chl*a*, *a*_g_(λ), and SPM at the cruises end. Chl*a* and *a*_g_(λ) were measured by using a Shimadzu UV2700 spectrophotometer (Shimadzu, Inc.)^38,39^. SPM was measured using the pre-combustion and weighing methods^40^. Spectral Evolution PSR-1100f (350–1050 nm) was used to measure spectral data containing the total water-leaving radiance (L_sw_), the sky radiance (L_sky_), and the radiance of the reference gray panel (L_p_), which radiances were used to calculate *R*_*rs*_(λ) with the following formula^41^:

1 $${R}_{{rs}}\left({\rm{\lambda }}\right)=\left[\left({{\rm{L}}}_{{\rm{sw}}}-{\rm{\rho }}\times {{\rm{L}}}_{{\rm{sky}}}\right)\times {{\rm{\rho }}}_{{\rm{p}}}\right]/\pi \times {{\rm{L}}}_{{\rm{p}}},$$

where ρ is Fresnel reflectance set as 0.028^42^, ρ_p_ is the reflectance of the gray panel (30%). Lastly, each band wavelength-center *R*_*rs*_(λ) was convolved according to the Spectral Response Function (SRF) of the MSI sensor.

**Table 2:** Lakes with recorded salinity field data from published papers for model training or independent cross-validation (CV).

Lake	LON	LAT	Number	Year	Reference	Usage
Daihai	112.69	40.57	12	2017	^26^	Training
Dalinor	116.64	43.29	18	2019	^27^	Training
Dalinor	116.64	43.29	10	2021	^28^	Training
Daihai	112.69	40.57	9	2020	^29^	Independent CV
Daihai	112.69	40.57	6	2020, 2023	^30,31^	Independent CV
Juyan	101.25	42.30	15	2020, 2023	^31,32^	Independent CV
Ulansuhai	108.85	40.96	6	2015–2019, 2023	^31,33^	Independent CV
Hongjiannao	109.89	30.09	4	2021, 2023	^31,34^	Independent CV
Hongjiannao	109.89	30.09	5	2016, 2019	^35,36^	Independent CV
Hulun	117.71	49.37	1	2023	^31^	Independent CV
Chagannaoer	114.91	43.42	2	2016, 2023	^31,37^	Independent CV
Dalinor	116.64	43.29	1	2016	^37^	Independent CV

### Sentinel-2 MSI image derived Rrs(λ)

A total of 976 Sentinel-2 MSI Level-1C images were downloaded from the Copernicus Data Space Ecosystem (https://dataspace.copernicus.eu/) during the period from March 2016 to October 2024. Each MSI tile covers approximately 100 × 100 km^2^ and the overpass is at 10:30 local time to minimize the effects of sunglint and cloud cover, and an orbital altitude is 786 km^43^. The available images for each year in spring, summer, and autumn were downloaded under cloudless or low-cloud (<10%) conditions. The downloaded MSI images were calibrated using a dark spectrum fitting (DSF) algorithm integrated into the Atmospheric Correction for OLI lite (ACOLITE) processor to derive *R*_*rs*_(λ). The DSF algorithm was designed for atmospheric corrections in coastal and inland waters and has been widely used^44,45^. The MSI image-derived *R*_*rs*_(λ) contains 11 bands with resampled spatial resolution of 10 m.

### Lake water extraction

Lake outline data from the Lake-Watershed Science Data Center (http://lake.geodata.cn) were used as initial boundaries for water extraction. The dataset contains accurate lake names, locations, and area properties. Vector lake outlines were interpreted based on China Brazil Earth Resources Satellite (CBERS) and Landsat-5 images, associated with digital elevation maps and lake chronicles^46^. Google Earth Engine (GEE) platform archived lake outlines and matched Sentinel-2 images to extract water by using the normalized difference water index (NDWI, Eq. (2)) and OTSU method^47^.

2 $${\rm{NDWI}}=\left({\rm{G}}-{\rm{NIR}}\right)/\left({\rm{G}}+{\rm{NIR}}\right),$$

where G is the green band (560 nm) and NIR is the near-infrared band (842 nm). Accurate estimation of lake salinity was difficult in aquatic plant waters due to the optical properties differing from normal waters and tending to cause high reflections in the infrared band. Therefore, combined with the Floating Algae Index (FAI, threshold of 0.03, Eq. (3)) to exclude aquatic plant waters in grass-type lakes (Ulansuhai Lake)^48^.

3 $${\rm{FAI}}={\rm{NIR}}-{\rm{R}}-\left[\left({\rm{SWIR}}-{\rm{R}}\right)\times \left({{\rm{\lambda }}}_{{\rm{NIR}}}-{{\rm{\lambda }}}_{{\rm{R}}}\right)/\left({{\rm{\lambda }}}_{{\rm{SWIR}}}-{{\rm{\lambda }}}_{{\rm{R}}}\right)\right]$$

where for the MSI sensor, the FAI calculate bands were adjusted to be the red band (R, 664 nm), NIR, and short-wave infrared band (SWIR, 1610 nm), with λ as the corresponding wavelength.

In addition, due to incorrect salinity estimates caused by bottom reflection and adjacency effects of mixed pixels in optically submerged lakes, possibly, a two-pixel buffer was created to minimize these influences^49^. Removed small patches (<3 pixels) and then calculated the lake area by ArcGIS 10.2 (WGS1984_UTM projection) in post-processing.

**Fig. 3:**
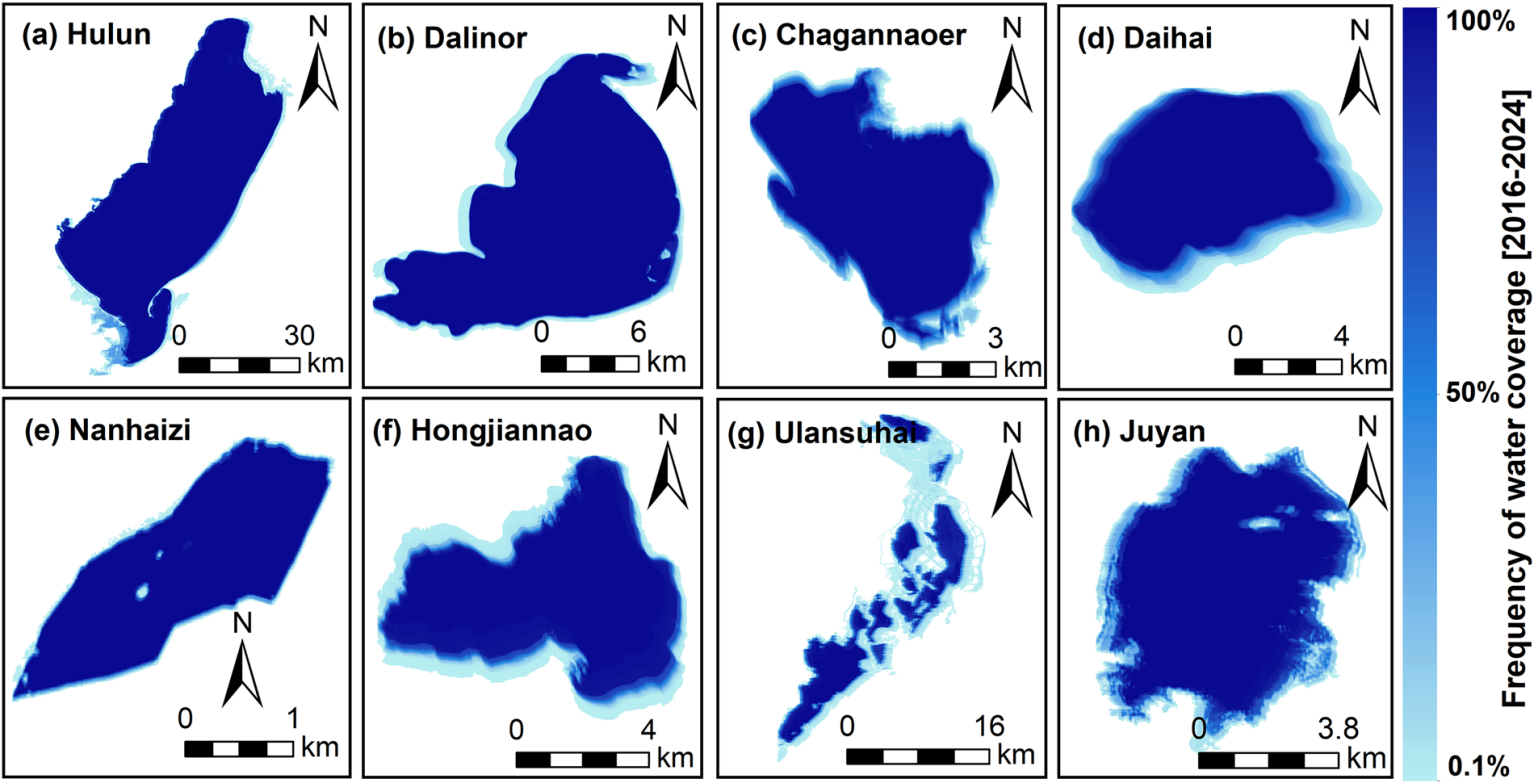
Water coverage frequency maps for eight lakes during 2016 to 2024.

This study generated water coverage frequency maps and detected the change rate of lake area by Mann-Kendall (M-K) test in eight lakes during 2016 to 2024^50^ (Fig. 3 and Table 3), and the M-K test was implemented by Python kernel. The constant water frequency threshold was defined as 95% to minimize errors due to cloud cover on the lake surface. The water coverage frequency in Daihai, Hongjiannao, Juyan, and Ulansuhai lakes has fluctuated more drastically compared to other lakes (percentage ≤ 65%); notably, the lake area of Daihai has significantly shrunk over the past nine years (change rate: −1.10 km^2^/year, *p* < 0.01). In contrast, Hulun, Dalinor, and Nanhaizi lakes have maintained relatively constant water boundaries (>84%) in the past nine years. Lake area of Hulun (13.98 km^2^/year, *p* < 0.01) and Nanhaizi (0.002 km^2^/year, *p* < 0.01) exhibited an increasing trend, while Dalinor (−1.01 km^2^/year, *p* < 0.01), Chagannaoer (−0.18 km^2^/year, *p* < 0.01), Daihai, and Juyan (−0.19 km^2^/year, *p* < 0.01) lakes decreased. Hongjiannao and Ulansuhai lakes did not indicate significant area changes (*p* > 0.27).

**Table 3:** Statistics on total water area, constant water area (water coverage frequency greater than 95%), percentage of constant water, and change rate of water area in each lake from 2016 to 2024.

Lake name	Total water area (km^2^)	Constant water area (km^2^)	Percentage (%)	Change rate (km^2^/year)	*p*-value
Hulun	2,313.77	1,953.62	84	13.98	*p* < 0.01
Dalinor	204.45	174.43	85	−1.01	*p* < 0.01
Chagannaoer	35.08	23.92	68	−0.18	*p* < 0.01
Daihai	62.28	39.67	64	−1.10	*p* < 0.01
Nanhaizi	3.35	2.85	85	0.002	*p* < 0.01
Hongjiannao	47.38	30.64	65	−0.05	*p* = 0.27
Ulansuhai	272.94	48.02	18	0.24	*p* = 0.51
Juyan	42.31	27.24	64	−0.19	*p* < 0.01

### Estimation of lake water salinity

One of the most widely applied and effective machine learning algorithms, XGB, was used to build the lake salinity model^51^. As an integrated learner based on decision trees of XGB that realizes gradient descent by residual iteration and adds a regularization term to prevent overfitting. Each tree contributes to the final prediction by a corresponding score based on the leaf node to which the input data belongs, and the final prediction is the sum of these scores across all trees in the ensemble. Good prediction accuracy and robustness of the algorithm have been demonstrated in the establishment of a retrieval model for the regression task of lake water quality parameters^52,53^.

The XGB salinity algorithm was implemented by the Python kernel with the sklearn library in this study. The main phases of salinity modeling included feature selection, model training, testing, and validation (Fig. 2). Thirteen input features were filtered by an important assessment algorithm already embedded in XGB, namely, *R*_*rs*_(443), *R*_*rs*_(497), *R*_*rs*_(560), *R*_*rs*_(664), *R*_*rs*_(704), *R*_*rs*_(740), *R*_*rs*_(842), NDWI, chromaticity angle (alpha), and lake area. The evolution of lakes is inevitably associated with changes in water volume and area, especially in arid and semi-arid regions, which can affect the water physicochemical properties (e.g., salinity)^18,54^. Alpha is a physical quantity related to the composition and inherent optical properties of the optical deep water, with the formula that can be found in Wang *et al*.^55^. The 229 matched pairs of salinity and *R*_*rs*_(λ) were randomly divided into 70% training set (N = 153) and 30% test collection (N = 76), depending on the size of *Dataset one* and the ratio frequently used for the XGB algorithm. The model arguments and structure were determined by training collections, and the model performance was evaluated through testing collections. In the training process, the *GridSearchSV* method was employed to determine the model hyperparameters; the number of trees was 500, the learning rate was 0.05, the maximum tree depth was 7, the subsample rate was 0.8, the regularization was 0.01, and the minimum child weight was set to 8. For evaluating the model performance, *Dataset one* was arbitrarily divided into five groups for implementing five-fold CV after defining the model’s structure^56^. The average statistical metrics of the five assessments were used to evaluate the XGB salinity model’s performance. Evaluation metrics consisted of the coefficient of determination (R^2^), root mean square error (RMSE), mean absolute error (MAE), mean absolute percentage error (MAPE), and bias (systematic error).

## Data Records

The IMSAL dataset, containing 928 salinity raster data and corresponding mean with standard deviation records at 10 m spatial resolution for eight lakes in Inner Mongolia over the past nine years, is available at the Zenodo repository (10.5281/zenodo.15849093)^57^. All raster data are stored in ‘TIF’ format and the data records are stored in Microsoft Excel xlsx files, which total about 29.7 GB. Each lake’s properties, including lake name, abbreviated name, location, elevation (m), districts, and types of climates, are catalogued in ‘*Lake_info.xlsx*’ (Table 4). Under the IMSAL dataset, it has eight subfolders named ‘*Lake_Name_SAL*’ and three tables. Each subfolder contains five data folders (Table 5). These data folders store the MSI *R*_*rs*_(λ)-retrieved salinity data, including daily salinity raster data (folder name: ‘*Abbreviation_Individual*’), average annual salinity (‘*Abbreviation_Annual*’), average intra-annual seasonal salinity (‘*Abbreviation_Intraannual_Season*’), average seasonal salinity (‘*Abbreviation_Season*’), and nine-year average salinity (‘*Abbreviation_Average*’). The attribute corresponding to ‘Abbreviation’ is available in ‘*Lake_info.xlsx*’. The raster name, mean, standard deviation (S.D.), MSI image identifier, and date for each raster scene are recorded in the statistics table with ‘*SAL_stat.xlsx*’ (Table 4). The metadata of the dataset is compiled in the table ‘*IMSAL_meta.xlsx*’. Table 5 lists the storage architecture of the IMSAL dataset, data format, raster spatial resolution, and temporal resolution.

**Table 4:** Property name and description of the IMSAL dataset.

Attribute	Description
Lake_Name	Adopted the lake name used in the lake outline dataset.
Abbreviation	Customized simplified names for each lake.
LON(DD)	Longitude coordinates of the lake’s center in decimal degrees.
LAT(DD)	Latitude coordinates of the lake’s center in decimal degrees.
Elevation (m)	The average elevation of the lake surface is derived from NASA Shuttle Radar Topography Mission (SRTM) Digital Elevation 30 m dataset.
Districts	Administrative district in which lake is located.
Types of climates	Dominant climate type at the lake location.
Individual	Daily lake salinity raster data, naming convention: ‘Abbreviation + YYYYMMDD_SAL’.
Annual	The average lake salinity raster data for each year, naming convention: ‘Abbreviation_YYYY’.
Intraannual_Season	The average lake salinity raster data for each season in a given year, naming convention: ‘Abbreviation + YYYY_season_average’.
Season	The average season lake salinity raster data from 2016 to 2024, naming convention: ‘Abbreviation_season_average’.
Average	The average lake salinity raster data from 2016 to 2024, naming convention: ‘Abbreviation_average’.
Raster_Name	Name of each lake salinity raster data.
SAL_Mean (ppt)	The mean values of the lake salinity raster data for each scene.
SAL_S.D. (ppt)	The standard deviation values of the lake salinity raster data for each scene.
Images_ID	Identifier of Sentinel-2 MSI images used.
Date	The date of the MSI images (YYYYMMDD).

**Table 5:** The storage architecture of the IMSAL dataset, data format, and the spatiotemporal resolution of raster data.

Folder name	Subfolder name	Filename	Format	Spatial resolution	Temporal resolution
Lake_Name_SAL	Abbrevation_Individual	Abbrevation + YYYYMMDD_SAL	TIF	10 m	Daily
	Abbrevation_Annual	Abbrevation + YYYY_average	TIF	10 m	Yearly
	Abbrevation_Intraannual_Season	Abbreviation + YYYY_season_average	TIF	10 m	Quarterly
	Abbrevation_Season	Abbreviation_season_average	TIF	10 m	Nine-year
	Abbrevation_Average	Abbreviation_average	TIF	10 m	Nine-year
		Lake_infor	xlsx	\	\
		SAL_stat	xlsx	\	\
		IMSAL_meta	xlsx	\	\

The attribute corresponding to ‘Lake_Name’ and ‘Abbreviation’ are available in ‘*Lake_info.xlsx*’. “\” denotes not applicable.

## Technical Validation

### Accuracy assessment of ACOLITE derived MSI *R*_*rs*_(λ)

**Fig. 4:**
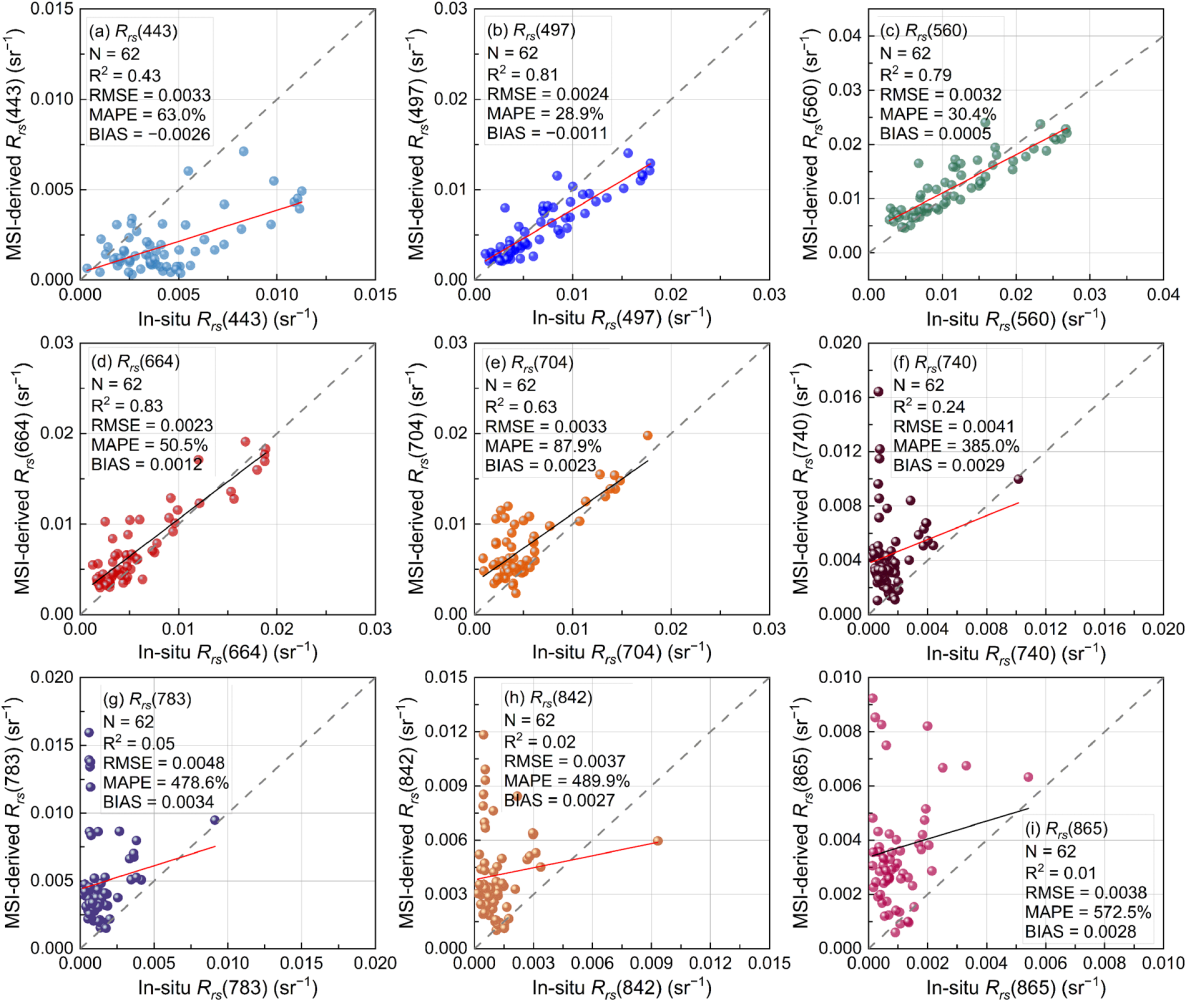
Scatterplot of *in situ R*_*rs*_(λ) and MSI-derived *R*_*rs*_(λ) using the ACOLITE processor.

Considered that accurate salinity estimation is affected by the performance of atmospheric correction, it was necessary to evaluate the consistency of MSI images-derived *R*_*rs*_(λ) with *in situ* measured *R*_*rs*_(λ) (Fig. 4).The MSI-derived *R*_*rs*_(λ) has the highest accuracy at 664 nm (N = 62, R^2^ = 0.83, and RMSE = 0.0023 sr^–1^) and outperforms the NIR band (704–865 nm) in terms of precision at visible wavelengths (497–664 nm). Overestimated *R*_*rs*_(λ) by AC processor in the infrared range absorbed by water where minor values are prone to error. Although the processors do not perform well at 783–865 nm, these wavelength bands were not the primary input features of the model. Therefore, the input features *R*_*rs*_(λ) involved in model training were reliable. The ACOLITE processor exhibits acceptable performance overall (RMSE < 0.0048 sr^‒1^, |bias| < 0.0034 sr^‒1^) and demonstrated it can be employed to derive *R*_*rs*_(λ) from MSI images (Table 6).

**Table 6:** Accuracy statistics of *in-situ* measurements *R*_*rs*_(λ) and ACOLITE-derived MSI *R*_*rs*_(λ).

Wavelength (nm)	R^2^	RMSE	MAPE (%)	BIAS
443	0.43	0.0033	63.0	−0.0026
497	0.81	0.0024	28.9	−0.0011
560	0.79	0.0032	30.4	0.0005
664	0.83	0.0023	50.5	0.0012
704	0.63	0.0033	87.9	0.0023
740	0.24	0.0041	385.0	0.0029
783	0.05	0.0048	478.6	0.0034
842	0.02	0.0037	489.9	0.0027
865	0.01	0.0038	572.5	0.0028

### Overall accuracy assessment

**Fig. 5:**
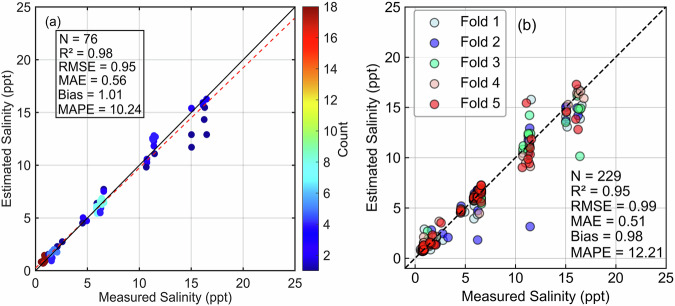
Scatterplot of XGB salinity model validation, (**a**) testing of 30% independent set, (**b**) five-fold cross-validation by using *Dataset one*.

The XGB salinity model performs with considerable accuracy (R^2^ = 0.98, RMSE = 0.95 ppt, MAE = 0.56 ppt, and MAPE = 10.2%) on the 30% independent testing set, as shown in Fig. 5a. Estimated salinity ranged from 0.5 to 18 ppt, which was in good consistency with measured values. But slightly underestimated lake salinity in the range of 15–18 ppt is possibly related to the constraints of the model boundaries. Five-fold CV demonstrated accuracy comparable to 30% independent testing (R^2^ = 0.95, RMSE = 0.99 ppt, MAE = 0.51 ppt, and MAPE = 12.2%), and the salinity derived from cross-validation was consistent with the extent of field measured, with only a few points underestimated (Fig. 5b). In general, the assessment results confirm that the XGB salinity model has good precision and robustness for salinity inversion based on MSI *R*_*rs*_(λ).

### Pixel-based statistical validation

**Fig. 6:**
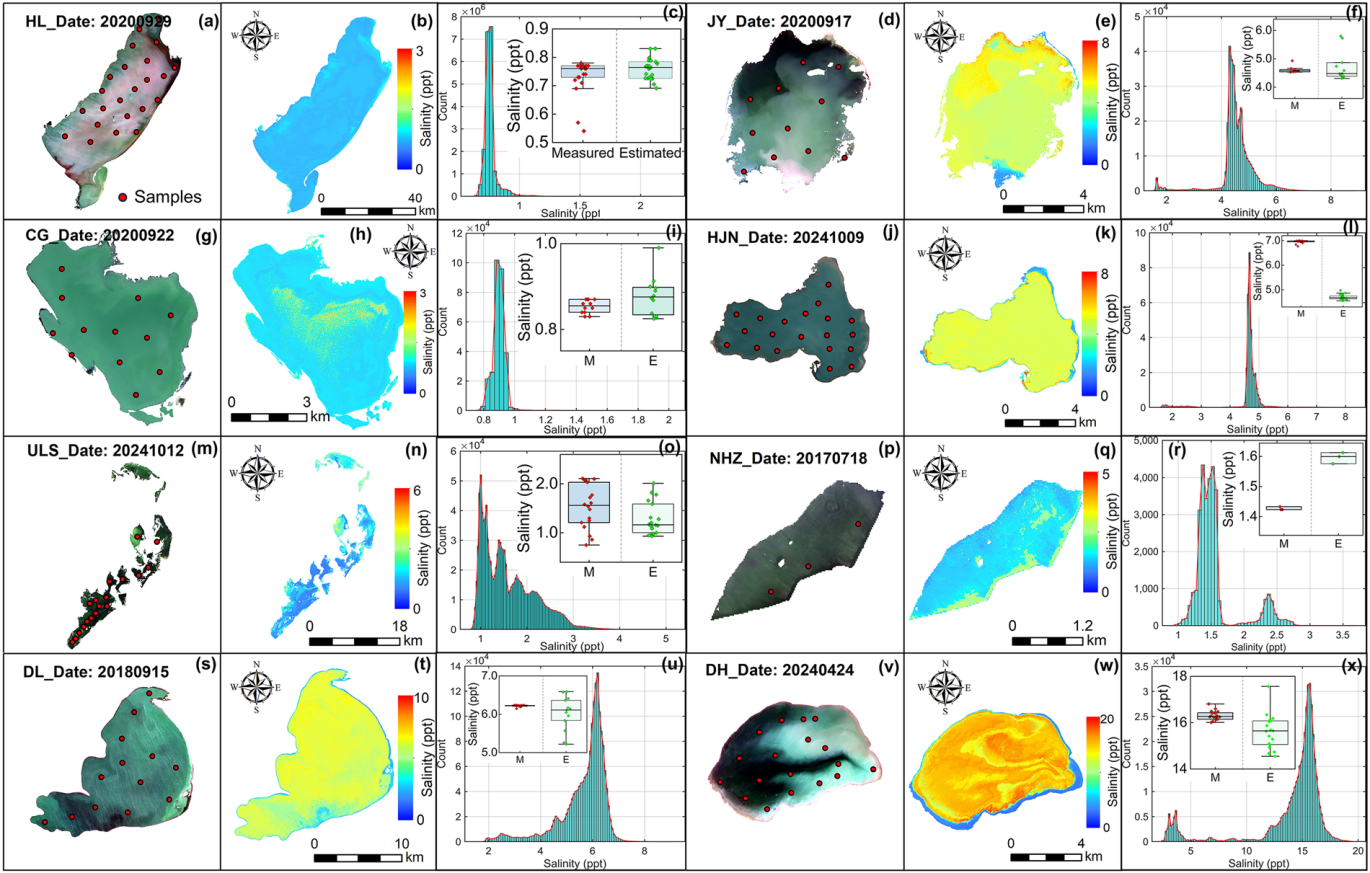
Nature color lake composited by MSI band (R, G, B), salinity raster data derived from MSI images by the XGB salinity model, histogram statistics, and box plots of salinity data.

Salinity raster data retrieved by concurrent satellite images was selected to histogram statistics for the lake-wide pixels (Fig. 6). The mode of estimated salinity can be visualized and reveal the outliers in the raster data by image histogram. The outliers are commonly distributed at both ends of the histogram with few pixels and abrupt value transitions. A single peak phenomenon was observed in the salinity histogram of most lakes and less variation within the lake (Fig. 6). It was consistent with the mode of salinity distribution measured in the field. The bimodal characteristics were found in a few lakes and with a scarcity of pixels on the sub-peak (Fig. 6r,x). The outlier pixels were observed in the Daihai Lake at the extent of 0–5 ppt due to being affected by mixed pixels in nearshore waters, probably, and not found in other lakes (Fig. 6x). Box plots display the mean and standard deviation of measured and estimated salinity with smaller deviations for most lakes. Overall, substantial outlier pixels were not observed in the salinity raster images and demonstrated good quality.

### Lake salinity independent validation

**Fig. 7:**
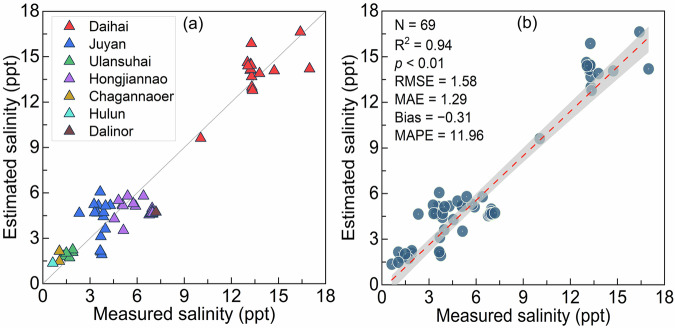
Scatterplot of estimated salinity through the XGB model versus the latest field and published literature-recorded measured salinity.

To objectively assess the reliability and science of the IMSAL dataset, independent validation was carried out by using *Dataset two*, which was not involved in model training and testing (Fig. 7). A total of 69 recent field-sampled and literature-recorded data were used for independent validation, which met a one-day matching interval to the salinity raster images. For historical salinity raster images, the validation density was not enough due to less matching data. The small size of *in situ* measurements from Lake Nanhaizi (N = 3) that have all been employed for model training and testing, they were not involved in independent validation (Fig. 7a). Field sampling of this lake will be expanded in future work. From the fitted line in Fig. 7b, the independent validation achieved a good precision (N = 69, R^2^ = 0.94, *p* < 0.01, and MAPE = 11.9%). Overestimation of salinity was a major manifestation of the XGB model error, especially in the range from 0 to 6 ppt, with more training samples from oligosaline-type lakes than brackish-type lakes, which boosted low-salinity predictions. But the XGB model slightly underestimated lake salinity by 6–9 ppt because of the few model overfittings caused by dense training data in this range (Fig. 5b). Note that there may be high uncertainty (RMSE = 1.58 ppt) in the estimation of salinity in freshwater lakes (<1 ppt) due to poor salinity spectral characteristics in freshwater lakes as a result of inadequate freshwater-salt mixing and low water salinity. Additionally, unavoidable systematic errors existing in fitting nonlinear relationships between salinity and *R*_*rs*_(λ) exacerbated uncertainty in freshwater lakes. Numerical comparisons of external data demonstrate again that IMSAL has science to it.

## Usage Notes

Effective monitoring of the water salinity is essential for preventing lake salinization and assessing water quality conditions. But regional spatiotemporal monitoring of lake salinity remains enough. The IMSAL dataset fills the deficiency to some extent and provides help for the public to understand the spatial patterns and trends in lake salinity. Variations of lake salinity in arid and semi-arid areas respond sensitively to climate, which means a chance to mine meteorological intelligence from the salinity dataset for climatologists. Note that this distinction can be made in conjunction with field data when the user requires in-depth research that carefully distinguishes between freshwater and brackish water, as the model has uncertainties in freshwater. The insufficient density of independent validation for historical salinity images inspires our future work, which needs to increase the sampling frequency or establish routine monitoring stations to collect long-term validation data. Model underestimation of salinity in the 15–18 ppt range may cause untimely monitoring of changes in water properties and seasonal patterns, resulting in delayed response measures. Potential correction strategies include expanding the model boundaries by adding training samples from high-salinity lakes or building a correction function to recalibrate for oligosaline lake salinity through field sampling. In addition, our established XGB model for rapid inversion of lake salinity based on remote sensing also offers technical insights for generating lake salinity datasets in other regions as well. Different from salinity data constructed using environmental data modeling and field measurements^53,58^, the XGB salinity model constructed based on remote sensing imagery in this study has the ability to historically reconstruct and iteratively update data. The complete procedure code for the building of the XGB salinity model and employed to MSI images was given in this study. If the user is interested in other lakes, the training samples can be extended from the current model to construct a practical model.

## Data Availability

Demonstration code for the construction, five-fold cross-validation, and application of the XGB salinity model is available at https://github.com/MingMDeng/SAL_inversion.git and should be accessed and edited using Python 3.11. Atmospheric correction of the Sentinel-2 MSI data was accomplished through the ACOLITE software (version 20221114.0). Extracted remote sensing reflectance and mosaic images in ENVI 5.3. Pixel-based manipulation and salinity data visualization in ArcGIS 10.2.
